# Impact of Fiber Diameter and Surface Topography of PCL Nanofibers on *Lacticaseibacillus rhamnosus* Biofilm Formation and Resistance

**DOI:** 10.1021/acs.jafc.5c16509

**Published:** 2026-05-12

**Authors:** Elisa Andreani, Vaclav Peroutka, Eva Kuzelova Kostakova, Ema Chudobova, Pavel Kejzlar, Sarka Hauzerova, Kristyna Havlickova, Marta Stindlova, Jana Jiresova, David Lukas, Simona Lencova

**Affiliations:** † 52735 University of Chemistry and Technology Prague, Faculty of Food and Biochemical Technology, Department of Biochemistry and Microbiology, Technicka 5, Prague 16628, Czech Republic; ‡ University of Insubria, Department of Biotechnology and Life Sciences, Via Jean Henry Dunant 3, Varese 21100, Italy; § 48261 Technical University of Liberec, Faculty of Science, Humanities and Education, Department of Chemistry, Studentska 1402/2, Liberec 46117, Czech Republic; ∥ 457957 Technical University of Liberec, Institute for Nanomaterials, Advanced Technology and Innovations, Department of Advanced Materials, Bendlova 1409/7, Liberec 460 01, Czech Republic; ⊥ University of Chemistry and Technology Prague, Faculty of Chemical Engineering, Department of Physics and Measurements, Technicka 5, Prague 16628, Czech Republic

**Keywords:** electrospun nanofibers, polycaprolactone, fiber diameter, surface topography, shish-kebab, probiotic biofilm, Lacticaseibacillus rhamnosus

## Abstract

Electrospun nanofibrous matrices are an emerging platform for probiotics. We evaluated polycaprolactone (PCL) nanomaterials, hypothesizing that fiber diameter and shish-kebab (*sk*) structures would increase biofilm formation and resistance. Four unique nanomaterials (PCL45, PCL45-sk, PCL80, PCL80-sk) were electrospun and tested with two *Lacticaseibacillus rhamnosus* strains (ATCC 9595, ATCC 53103). Biofilms formed after 48 h and 8 days were analyzed for biomass (CFU/cm^2^), structure (SEM), and metabolic activity (MTT). PCLs enhanced biofilm formation compared with polystyrene (reaching up to 8.7 ± 0.2 log_10_(CFU/cm^2^) for *L. rhamnosus* ATCC 9595 after 48 h); prolonged cultivation revealed strain-specific responses, with fiber diameter and *sk* significant impact. PCL-supported biofilms showed a good response to a short-term challenge of low pH (2, 4), with the minimal 97.9 ± 3.7% survival rate, and a preliminary, strain-dependent improvement in resilience to *Staphylococcus aureus*, mostly for *L. rhamnosus* ATCC 9595. This identifies PCLs as promising, adjustable carriers for probiotic biofilms.

## Graphical abstract

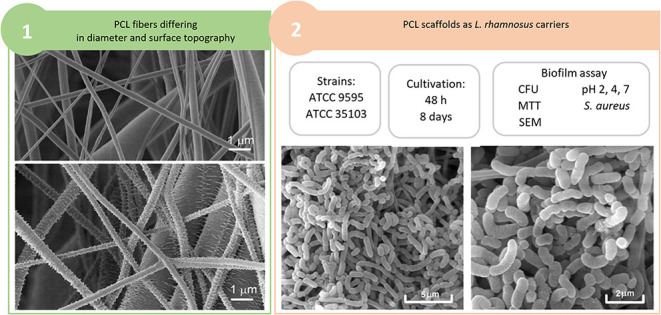

## 1 Introduction

Currently, probiotics represent a rapidly expanding global industry worth billions of dollars and rank among the most widely consumed dietary supplements worldwide.^1^ Probiotics are defined as “live microorganisms which, when administered in adequate amounts, confer a health benefit on the host”.^2^ Commonly, most of the bacterial probiotic strains used in the food industry and medicine belong to *Lactobacillus* and *Bifidobacterium* species.^3^ Probiotic bacteria are believed to benefit human health through three primary mechanisms. First, certain probiotics can inhibit or exclude pathogens, either directly or by influencing the commensal microbiota. For instance, lactobacilli exhibit inhibitory effects against various pathogens, including *Escherichia coli*, *Salmonella species*, *Listeria monocytogenes*, *Staphylococcus aureus*, and other bacteria.^4–7^ Second, specific probiotic strains enhance epithelial barrier function by modulating signaling pathways leading to effects like mucus induction or improved tight junction function. Third, most probiotics modulate host immune responses, exerting strain-specific local and systemic effects.^3^

Bacteria are able to adapt to various lifestyles in their natural habitats, ranging from single planktonic cells to biofilm communities and biofilm-dispersed cells. Biofilms are surface-associated communities of microorganisms embedded in a self-produced extracellular matrix of extracellular polymeric substances (EPS), which exhibit a much higher level of organization compared with planktonic cells.^8–10^ Unlike planktonic or passively immobilized cells, biofilm-embedded bacteria benefit from the protective properties of the EPS, which enhance their resistance to extreme pH, temperature fluctuations, and toxic agents. Moreover, cells within biofilms remain metabolically active for longer time durations. These advantages make biofilms a more suitable starting material for the production of bacterial-derived products compared with planktonic or immobilized cells.^11^

In biotechnology, biofilm carriers are being developed to enable large-scale production, long-term stability, and resistance to external factors. In recent years, the use of electrospun nonwoven nanomaterials for these applications has seen significant growth.^12^ While the impact of organic nanofibers on eukaryotic cells has been extensively researched in tissue engineering and nanobiotechnology, their use in functional bacterial cultures has been much less explored, leading to the need for further studies on this topic to be conducted.^13^ Similarly, the formation of bacterial biofilms on nanofiber membranes and the potential application of biofilm-integrated nanofiber membranes are still largely uncharted.^14^ To date, only a few studies have explored the use of polymeric fibers as microbial cell carriers, but generally, no proper controls have been used, or limited quantitative data of cell attachment have been provided to properly estimate the cell attachment level achieved.

Among various polymers suitable for the electrospinning technique, polycaprolactone (PCL) has attracted considerable attention due to its biodegradability and its susceptibility to degradation by diverse bacterial species. PCL has received approval from both the Food and Drug Administration and Conformité Européenne marking for use in various drug delivery systems and medical devices, though relatively few have been commercialized or applied in clinical research.^15^ Unlike other nanomaterials, studies focusing on the morphology of PCL fibers, commonly used in medical and biotechnological applications, have yielded inconsistent conclusions. Tamayo-Ramos et al.^16^ and De Cesare et al.^17^ investigated PCL microfibers and nanofibers with varying fiber diameters but reported contrasting results regarding the influence of fiber diameter on biofilm formation. These results highlight the need for further investigation into the influence of fiber diameter on biofilm formation.

In addition to the influence of basic morphological parameters, nanofiber materials made of PCL can be further surface-treated in very interesting ways. It is possible to create variously structured surfaces, such as porous fibers^18^ or fibers with a so-called shish-kebab (*sk*) structure.^19^ The *sk* fiber structure typically exhibits lamellar protrusions oriented perpendicular to the fiber axis. Increasing the roughness of the nanofiber surface, i.e., increasing the specific surface area of the nanofibers to obtain *sk* fibers, is possible with PCL nanofibers by a simple process of dipping or spraying the nanofibers with diluted PCL solutions in imperfect solvents such as pentyl acetate^20^ or a mixture of acetic acid and water,^21^ etc. This creates interesting surface-structured porous nanomaterials.

In the present study, we investigated the potential of PCL nanomaterials to promote biofilm formation by *Lacticaseibacillus rhamnosus* (formerly *Lactobacillus rhamnosus*) strains—a well-studied lactic acid bacterium that exhibits the desirable characteristics commonly found in conventional probiotic strains.^7,22^ This study builds on our previous work on electrospun PCL nanofibers, where we evaluated the effect of fiber diameter on biofilm formation and bacterial cell retention for opportunistic pathogens *E. coli* and *S. aureus*.^23^ Here, we extend that platform by introducing postprocessed *sk* surface topographies and shifting the biological focus to probiotic *L. rhamnosus*, complemented by a broader microbiological evaluation by testing the essential research hypotheses specified below.

The aim of this study is to provide basic insight into how PCL nanofiber structure affects *L. rhamnosus* biofilm formation and biofilm durability, thereby informing the use of nanofiber carriers for probiotic biofilms. The following hypotheses were tested: **H1** “PCL nanomaterials enhance biofilm formation compared to polystyrene (PS) reference”, **H2** “Fiber diameter plays a significant role in promoting biofilm formation”, and **H3** “Shish-kebab surface morphology of nanofibers influences the biofilm formation”. Unique PCL nanofibers with varying fiber diameters and surface morphologies (smooth/*sk* structures) were analyzed. The resulting biofilms were exposed to challenging conditions, including different pHs and microbial contamination, to evaluate their resilience in comparison to conventional biofilm formed on a reference material, PS. Statistical analyses were conducted to validate these findings. Owing to the novelty of this work, the results offer valuable insights for future studies on the interplay between nanomaterials and probiotic biofilm systems.

## 2 Materials and Methods

### 2.1 Nanomaterials Preparation and Characterization

The following two basic polymer solutions were created for electrospinning: (i) 16 wt % PCL with a molecular weight of 45 000 g/mol (PCL45; Purasorb PC08, Corbion, Netherlands) in a chloroform/ethanol solvent system 8:2 by weight; (ii) 10 wt % PCL with a molecular weight of 80 000 g/mol (PCL80; Sigma-Aldrich, Germany) in a chloroform/ethanol solvent system 8:2 by weight. Unlike chloroform, ethanol is not a perfect solvent for PCL, but it causes slower evaporation in the solvent system, thus allowing for longer fiber elongation during the spinning process.

Electrospinning, i.e., the production of nanofiber layers, was performed on a device for continuous needleless electrospinning using direct current NS 1S500U (Elmarco, Czech Republic), supplemented with an air conditioning unit for precise adjustment of relative humidity and ambient air temperature NS AC150 (Elmarco, Czech Republic). The parameters of the spinning process were as follows: electrode distance 180 mm; polymer solution feed rate, i.e., polymer solution carriage speed 0.5 m/s; orifice in feeding insert diameter 0.7 mm; ambient air humidity 30% RH and temperature 22 °C; electrical voltage at spinning wire electrode +40 kV; electrical voltage at collector wire electrode −10 kV, substrate material, spunbond nonwoven fabric with an areal weight of 30 g/m^2^ (PFNonwovens, Czech Republic); substrate nonwoven fabric withdrawal speed for PCL45 was 25 mm/min and for PCL80 was 10 mm/min.

Samples of nanofiber layers PCL45 and PCL80 with a final basis weight of 40 g/m^2^ were separated from the substrate nonwoven fabric and stretched onto a circular bamboo frame with a diameter of 15 cm. A diluted solution of PCL80 with a concentration of 1 wt % in pentyl acetate was prepared. The samples were sprayed with this solution so that they were completely wet. Excess liquid was then removed with paper towels and the samples were left to dry while stretched on the frames in a fume hood at standard laboratory conditions. The amount of diluted solution applied in the post-process treatment onto the nanofibrous layers was approximately 100 g/m^2^. The dry samples (PCL45-sk and PCL80-sk) were removed from the frames and cut to the required sample sizes for subsequent evaluation. The samples were scanned in detail using a scanning electron microscope Zeiss Ultra Plus equipped with a Schottky cathode (Zeiss, Germany). The samples were scanned on both sides and at least ten different locations, particularly at magnifications of 10 000× and 25 000×. The samples were first coated with a 2 nm thick conductive layer of platinum (sufficient for low currents of 1–2 pA and a voltage of 1 kV), the image does not drift at all, and the deposited layer is “invisible”, so there is no chance of distortion in the measurements (even for 25k magnification, the layer thickness is less than one pixel). It was not possible to scan the samples without a conductive layer, as this was necessary to prevent noise and image drift caused by charging the sample. SEM images were used to determine the estimated diameter of fibers in the nanofiber layers and also to estimate the size of surface structures on modified fibers using FIJI/ImageJ (NIH) software. After postprocessing, cone-shaped protrusions were observed on the surface of PCL45-sk fibers, whereas lamella-shaped protrusions were detected on PCL80-sk fibers. For PCL45-sk, the cone base length, cone height, and the characteristic distance between adjacent cones were quantified. For PCL80-sk, the characteristic spacing between neighboring lamellae was measured. All measurements were performed using high-magnification SEM images (25 000×). The principles of the individual measurements are illustrated in Figure 1.

**Figure 1:**
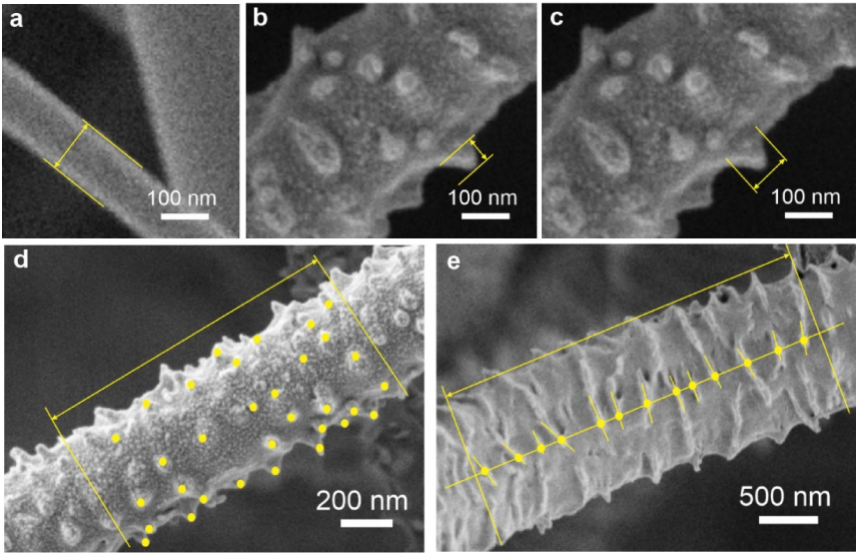
Measurement of basic smooth fiber diameter for PCL45 and PCL80 (a); linear characteristics of cones on PCL45-sk—height (b), base length (c), characteristic cone spacing (d); and characteristic spacing between lamellae on PCL80-sk (e). The figure schematically demonstrates the procedures applied for determining a) the diameter of smooth PCL45 and PCL80 fibers, b–d) the linear descriptors of cone-shaped surface protrusions on PCL45-sk fibers (cone height, base length, and characteristic cone spacing), and e) the characteristic spacing between lamellae on PCL80-sk fibers. This figure is included solely to clarify the measurement approach and the stereological and geometrical principles used for quantifying fiber diameter and surface structural parameters. It is not intended for morphological comparison between different materials or fiber types.

To determine the characteristic distance between cones on PCL45-sk fibers, the number of visible cone peaks was counted along a defined fiber length, ensuring that no peaks were counted twice in cases where the fiber surface was partially visible from the opposite side (Figure 1c). This approach is based on a stereological principle. Knowing the fiber diameter, the unfolded surface area of one-half of the cylindrical fiber can be estimated. From this surface area and the number of cones present, the average surface area per cone can be calculated. The characteristic distance between cones is then defined as the square root of this average area. For PCL80-sk, the average spacing between neighboring lamellae was estimated using a test line placed along the central axis of the fiber. The number of lamella intersections with this line was counted over a defined fiber length (Figure 1d).

### 2.2 Bacterial Cultivation

The bacterial strains *L. rhamnosus* ATCC 9595 (eq CCM 1828; LR1), *L. rhamnosus* ATCC 53103 (eq CCM 7091; LR2), and *Staphylococcus aureus* ATCC 25923 (CCM 3953; SA) were obtained from the Czech Collection of Microorganisms (CCM, Czech Republic). *L. rhamnosus* ATCC 53103 was originally isolated from the intestinal tract of a healthy human in 1983. Clinical studies have safely tested it in a wide range of individuals, from preterm infants to the elderly, with doses of up to 100 billion CFU/day and no serious adverse events reported.^24^ *L. rhamnosus* ATCC 9595 is a strain with probiotic potential.^25^ *S. aureus* ATCC 25923 is an international standard reference strain for antibacterial susceptibility testing.

The pure cultures of *L. rhamnosus* were grown in De Man–Rogosa–Sharpe broth (MRS) and on MRS agar (Merck, Germany) at 37 °C for 48 h, mixed with 25% glycerol, and stored in a freezer (−80 °C). Prior to the use, suspensions were recultivated by inoculation into sterile MRS and incubation at 37 °C for 48 h, followed by centrifugation (5 min, 5000 *g*) and resuspension of the obtained pellet in sterile MRS. Then, 24 h grown suspensions were used in biofilm assays. Pure culture of *S. aureus* was stored in tryptone soy broth (TSB, Merck, Germany) similarly to *L. rhamnosus*, grown in TSB and on TS agar (TSA, Merck, Germany) at 37 °C for 24 h, and directly used in the contamination assay.

### 2.3 Biofilm Formation

Sterile (30 min under UV light) PCL nanomaterials of size 1 × 1 cm were employed as biofilm carriers. A single piece of PCL was placed in a sterile 12-well microtiter plate (Greiner Bio-One, Austria) containing 2 mL of bacterial suspension (either LR1 or LR2) in MRS with the optical density of 0.5 McF (McFarland; McF standard is a reference turbidimetric unit of optical density used to estimate the concentration of bacterial cells in a suspension; 0.5 McF corresponds to approximately 1.5 × 10^8^ CFU/mL for *L. rhamnosus*). Samples were cultivated at 37 °C for 48 h or 8 days (with regular MRS medium change to keep a constant nutrient level every 48 h) under aerobic conditions. Control biofilms were formed under identical conditions on a reference material, PS, the material composing the microtiter plates.

### 2.4 Determination of Viable Biofilm-Forming Cells

Quantitative analysis of the formed biofilm was performed according to Lencova et al.^23^ PCL nanomaterials with formed biofilms were washed three times by soaking them into sterile saline solution (0.9% solution of NaCl in distilled water). Control wells were washed three times with sterile saline solution. Then, 1 mL of sterile saline solution was added to all wells. Plates were sonicated for 3 min in a sonication bath (Polsonic, Poland; conditions: frequency 40 kHz, power 150 W) at room temperature to uniformly release biofilm-forming cells. The obtained suspension was decimally diluted up to the sixth dilution. Droplets of 20 μL of individual dilutions were deposited on the surface of MRS agar. Following cultivation at 37 °C for 48 h under aerobic conditions, CFU/cm^2^ were determined for both control and nanomaterial-containing wells.

### 2.5 MTT Assay

The MTT assay was performed according to Stindlova et al.^26^ to assess metabolic activity, based on the reduction of MTT to purple formazan by metabolically active cells. Under these conditions, absorbance was used as a comparative indicator of bulk cellular redox activity rather than as a direct measure of viability. Plates were prepared as described in Section 2.3. After removing the supernatant, wells were rinsed twice with 1x phosphate-buffered saline (PBS), and nanomaterials were washed by immersion in 1x PBS. A glucose solution (58 mg/mL in 1x PBS) was added at 320 μL per well. Subsequent steps were conducted under limited light exposure. MTT solution (1 mg/mL) was then added at 280 μL per well and mixed by pipetting. Plates were wrapped in aluminum foil and incubated at 37 °C for 2 h. After incubation, the MTT-glucose solution was discarded, wells were rinsed once with 1 mL of washing solution, and plates were incubated for an additional 2 h at 37 °C to solubilize the formazan crystals. The contents were mixed by pipetting (three repetitions), and 100 μL of the suspension was transferred to a prewarmed sterile 96-well plate (37 °C). Absorbance was measured at 595 nm following plate heating at 45 °C for at least 20 min.

### 2.6 Short-Term pH Resistance Assay

Plates were prepared as described in Section 2.3, and biofilms were cultivated for 48 h. After removing the supernatant, biofilm-containing wells (including those with nanomaterials) were treated with 2 mL of a specific solution at pH 2, 4, or 7 (prepared by a controlled addition of HCl, adjusted with NaOH, and measured with a calibrated pH meter (Radiometer Analytical, France)) and incubated at room temperature for 10 min as recommended by Hu et al.^8^ Wells were then washed three times with sterile saline solution. Subsequently, 1 mL of sterile saline solution was added to each well, followed by 3 min of sonication. The resulting suspensions were serially diluted up to the sixth dilution, and 20 μL of each dilution was plated on MRS agar. After 48 h of aerobic incubation at 37 °C, CFU/cm^2^ and the percentual survival rate were determined for both control and nanomaterial-containing wells across the different pH conditions. In the context of this short-term pH challenge, the criterion for classifying biofilms as “highly resistant” was 80%, in line with the literature.^27^ This assay was designed as a simplified short-term acid challenge reflecting acute exposure to fasted-state gastric acidity^28^ rather than a full simulation of gastrointestinal transit.^29^

### 2.7 Antimicrobial Assay

A concentrated inoculum of both *L. rhamnosus* strains was used without adjustment to a specific optical density (OD). Each MRS agar plate was conceptually divided into two halves, and 20 μL of the concentrated inoculum was applied to each side. The plates were incubated aerobically at 30 °C for 72 h. A suspension of SA was prepared from refrigerated colonies grown on TSA plates. 1 mL of a 1 McF (corresponding to approximately 3.0 × 10^8^ CFU/mL) SA suspension was added to 100 mL of TSB supplemented with 7 g/L of agar. The agar plates containing *L. rhamnosus* strains were overlaid with a thin layer of the semi-solid TSB-agar medium containing SA. Plates were then incubated aerobically at 30 °C for 48 h. Inhibition zones were evaluated by measuring their diameters.

### 2.8 Contamination Assay

Plates for the contamination assay were prepared as described in Section 2.3; biofilms were formed for 48 h or 8 days. After removing the supernatant, biofilm-containing wells (including those with nanomaterials) were exposed to 1 mL of SA suspension (0.5 McF, corresponding to approximately 1.5 × 10^8^ CFU/mL) and incubated aerobically at 37 °C for 24 h. Wells were then washed three times with sterile saline solution, followed by the addition of 1 mL of sterile saline solution and 3 min of sonication. The suspensions were serially diluted up to the sixth dilution, and 20 μL aliquots of each dilution were plated on MRS agar for *L. rhamnosus* and Baird–Parker agar (Merck, Germany) for SA. CFU/cm^2^ and the percentual representation of SA were determined after 24 h, and *L. rhamnosus* after 48 h of aerobic incubation at 37 °C. According to the short-term pH resistance assay, a survival rate >80% was considered high, 50–79% moderate, and <50% low.

### 2.9 Scanning Electron Microscopy

Qualitative analysis of biofilms formed on PCL nanomaterials was performed as described in Lencova et al.^23^ and Peroutka et al.^30^ PCL nanomaterials were gently rinsed with 1x PBS and fixed in frozen ethanol (Penta, Czech Republic) for 15 min. After dewatering the samples (gradually increasing ethanol concentration from 60% to 99.8%), the PCLs were dried at laboratory temperature for at least 24 h. Before analysis, the nanomaterials were coated with gold (10 nm) and analyzed using SEM Tescan Mira 3 LMH (Tescan, Czechia) with a Schottky cathode at 15 kV accelerating voltage.

### 2.10 Statistical Analysis

All experiments were performed in at least three biological and technical replicates. Data analysis was performed in the R programming language in the RStudio program according to Peroutka et al.^30^ Data normality was verified using the Shapiro-Wilk test, data analysis and comparison were done using an unpaired two-sample *t*-test and multiple comparisons by one-way analysis of variance (ANOVA) at a significance level of α = 0.05. If a significant result was found, Tukey Honest Significant Differences (Tukey HSD) was calculated to perform multiple pairwise comparisons of groups’ means.

## 3 Results and Discussion

### 3.1 Prepared PCL Nanomaterials

Basic electrospun nanofiber materials with smooth fibers PCL45 and PCL80 and surface-modified materials PCL45-sk and PCL80-sk are shown in Figure 2. The diameters of the basic smooth fibers PCL45 and PCL80 are shown in the histograms in Figure 3. The average fiber diameters and corresponding 95% confidence intervals are 373 ± 73 nm for PCL45 and 1192 ± 156 nm for PCL80.

**Figure 2:**
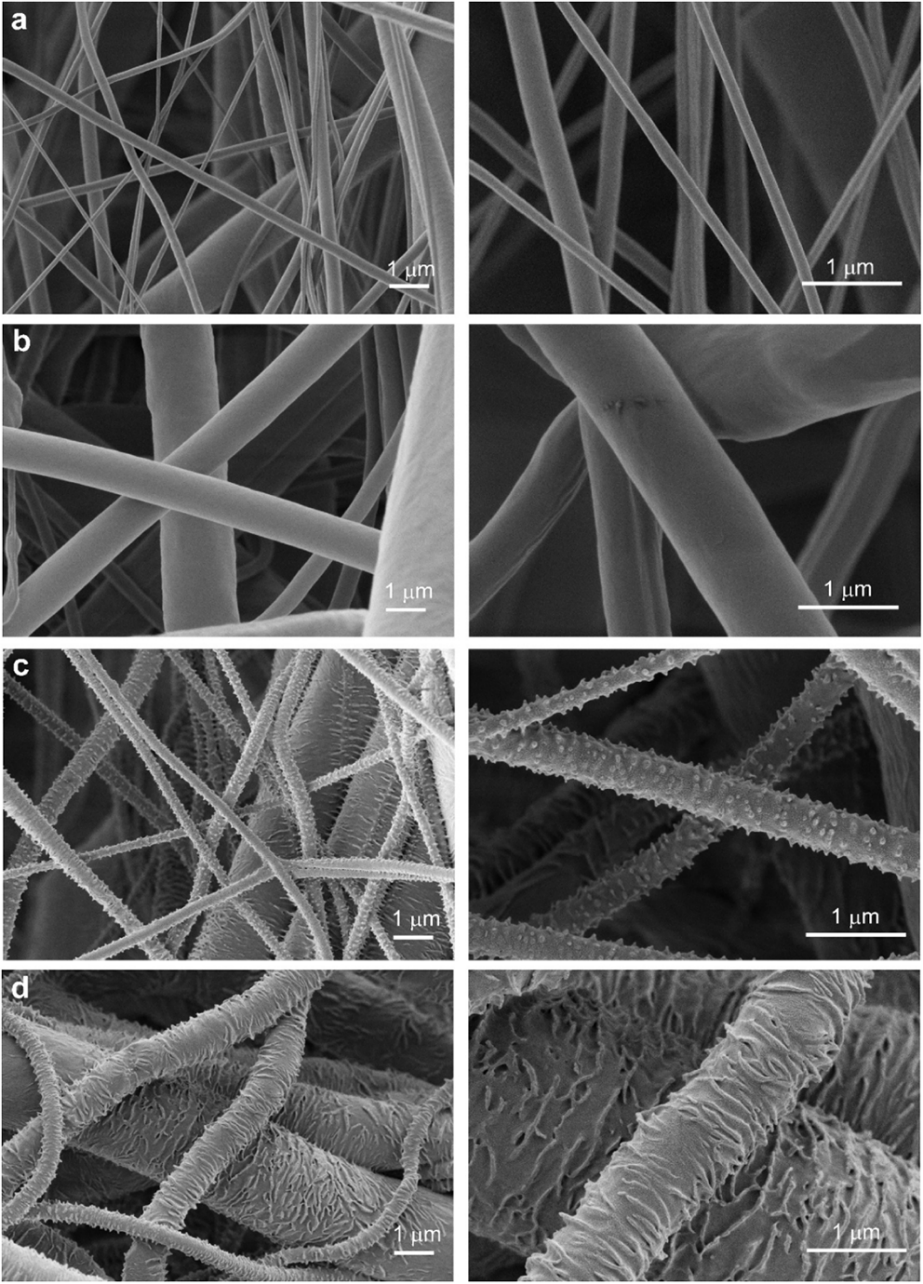
Examples of SEM images of: a) PCL45; b) PCL80; c) PCL45-sk; d) PCL80-sk at two different magnifications to clearly introduced the surface structure changes after the postprocess treatment by dilute PCL80 solution. The PCL45-sk with cone-shaped protrusions and PCL80-sk with lamella-shaped protrusions.

**Figure 3:**
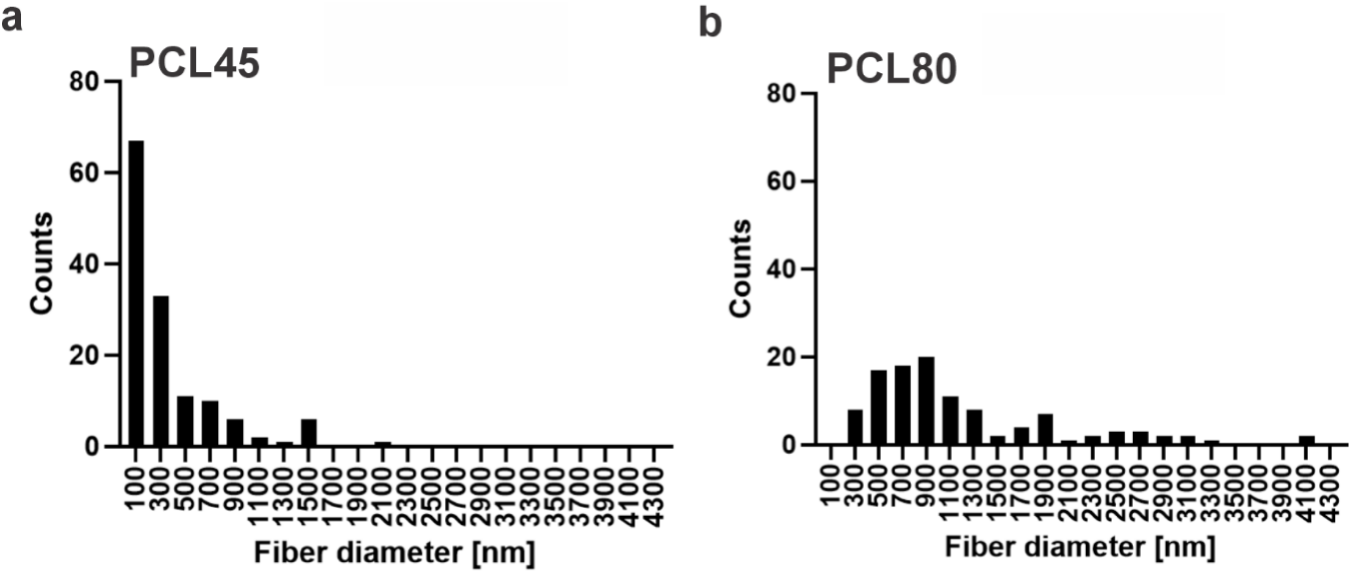
Histograms of fiber averages for basic electrospun materials PCL45 (a) and PCL80 (b).

However, both smooth electrospun materials exhibit a relatively broad fiber diameter distribution that deviates from normality and displays a pronounced multimodal character, as indicated by the qualitative inspection of the histograms revealing multiple local maxima. Therefore, the above-mentioned values (the average basic smooth fiber diameters and the confidence intervals) should be considered approximate, and it is the histograms that provide an accurate picture of the structure in question. No additional formal statistical tests for normality were applied, as the diameter distributions were intended primarily for descriptive characterization. This behavior can be attributed to the complex jet dynamics inherent to the electrospinning process, together with the combined influence of molecular weight-dependent chain entanglement density,^31^ polymer concentration, and selective solvent evaporation in a mixed good/nonsolvent system, where chloroform acts as a good solvent and ethanol as a nonsolvent.^32^ The presence of ethanol may induce local fluctuations in solvent quality, leading to transient phase instabilities and spatial variations in viscoelastic properties and solidification kinetics during jet stretching,^33^ which in turn can promote the coexistence of multiple fiber diameter populations.^34^ It is well established that the fiber diameter distribution of electrospun PCL is highly sensitive to the choice of solvent system,^35^ and analogous heterogeneous nanofiber morphologies formed from chloroform–ethanol mixtures have also been reported for other aliphatic polyesters, such as poly­(lactic acid).^36^

The SEM images clearly show that postprocessing of PCL45-sk (Figure 2c) resulted in cone-shaped protrusions on the surface of the fibers, while postprocessing of PCL80-sk (Figure 2d) resulted in longitudinal lamellae-shaped protrusions on the surface of the fibers. To the best of the authors’ knowledge, we are not aware of published reports describing comparable conical nanostructures on electrospun PCL fibers. The physicochemical mechanisms governing their formation therefore remain insufficiently understood and are currently under systematic investigation. While for lamellae-shaped protrusions, the mechanism of their formation is described in the literature as the mechanism of formation of a shish-kebab nanofibrous structure.^37^

For a more detailed description of the results obtained in this study, it can be stated that for fibers with smaller diameters, the lamellae are oriented almost perpendicular to the fiber axis, but as the diameter of the base fibers increases, the lamellae become multidirectional. The measured linear characteristics of the cones on the surface of the modified PCL45-sk fibers (average and 95% confidence interval) were: height 98 ± 8 nm, base length 114 ± 12 nm, and the characteristic cone spacing was estimated to be 174 ± 17 nm. For PCL80-sk, the characteristic spacing between lamellae (average and 95% confidence interval) was 283 ± 35 nm.

### 3.2 Influence of PCL Fiber Diameter and Surface Morphology on Biofilm Formation and Metabolic Activity

PCL nanofibers were selected here as biodegradable, nonantibacterial carriers for controlled probiotic biofilm cultivation relevant to food/nutraceutical processing. Given the inconsistent conclusions reported in studies investigating the role of PCL fiber morphology in biofilm enhancement, we evaluated four types of PCL nanomaterials differing in fiber diameter and surface morphology. To understand the influence of PCL on biofilm formation, the hypotheses **H1**, **H2**, and **H3** were systematically tested.

To evaluate the effects of both the PCL polymer and the morphology of the produced nanomaterials, experiments were performed at two cultivation durations: 48 h and 8 days, reflecting potential variations in biological system behavior under varying conditions. The 48 h period represents the standard time for stable biofilm formation, enabling comparison with other studies. For potential industrial applications in probiotic biomass/biofilm production and storage, however, prolonged cultivation with regular medium addition is also relevant, as it reflects potential yield and maximizes the cultivation’s capabilities. Therefore, an 8-day period was included. Differences in trends between short- and long-term experiments were expected, as biofilms may behave differently under extended nutrient supply than during short-term growth.

After 48 h cultivation, both LR1 and LR2 formed biofilm on all tested nanomaterials (Figure 4A), supporting previous reports on the biofilm-forming ability of *L. rhamnosus* species.^3,7^ For both tested strains, biofilm formation was enhanced (Tables S1A and S1C) on all tested nanomaterials compared to PS, reaching up to 8.7 ± 0.2 log_10_(CFU/cm^2^) for LR1 on PCL80 after 48 h. These findings align with previous reports showing that, under defined experimental conditions, PCL-based nanomaterials can support or enhance microbial biofilm formation, as demonstrated for *E. coli*, *Pseudomonas putida*, *Brevundimonas diminuta*, and *Sphingobium fuliginis*.^16,38^ Regarding the role of fiber morphology, no significant difference was observed between PCL45 and PCL80 for both strains, indicating no impact of fiber diameter, nor between smooth and *sk* fiber morphology, pointing out the insignificance of the surface structure of these materials for short-term biofilm cultivation (Tables S1B and S1D). These results contradict the findings of De Cesare et al.,^17^ highlighting the critical role of fiber diameter in facilitating biofilm development. However, several variations between our study and the study conceived by De Cesare should be mentioned. Specifically, nanomaterial preparation (even though differing in only several details, such as conditions applied, temperature, etc., while using a similar solvent system and electrospinning process) and resulting fiber diameter (less than 100 nm in the study of De Cesare, while our average tested fiber diameter ranged from 373 ± 73 nm for PCL45 to 1192 ± 156 nm for PCL80) varied, as well as tested bacterial strains (*Burkholderia terricola*) and microbiological assays reflecting the tested microorganism growth requirements. Therefore, the findings of individual studies are not fully comparable, underscoring the need for further research to validate the applicability of specific nanomaterials for defined purposes.

**Figure 4:**
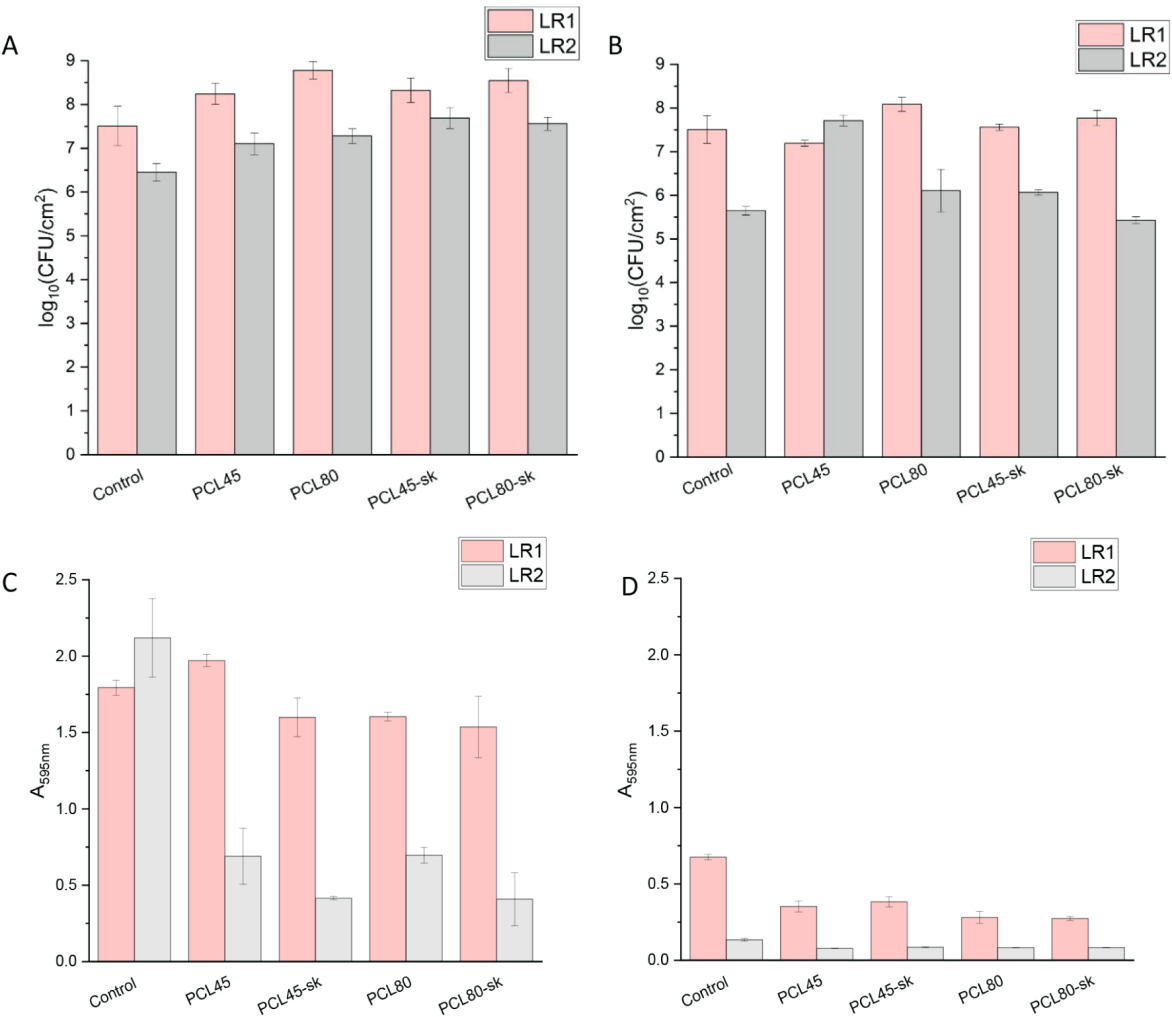
Quantitative representation of the biofilm formation and metabolic activity of biofilm-forming cells associated with smooth (PCL45, PCL80) and *sk* (PCL45-sk, PCL80-sk) nanomaterials after 48 h: A) CFU/cm^2^, C) MTT assay, and 8 days: B) CFU/cm^2^, D) MTT assay. Control = bacterial strains alone.

These experiments were followed by prolonged incubation of the bacteria with nanomaterials for up to 8 days (Figure 4B). Both strains formed matured biofilms, reaching up to 8.1 ± 0.2 log_10_(CFU/cm^2^) for LR1 on PCL80, which together with the 48-hour cultivation results indicates that both the material and bacterial species have a finite capacity for biofilm formation, as total biomass cannot increase indefinitely even if nutrients are regularly added. Prolonged cultivation slightly diminished the PS–PCL and intermaterials differences, with strain-specific response observed. For LR1, two materials (PCL45-sk and PCL80) influenced biofilm formation significantly (Table S2A and S2C); for LR2, all tested materials had a significant impact (Table S2B and S2D). Analysis suggests that compared to short-term cultivation, fiber diameter appeared to be a decisive factor, consistent with previous studies.^12,23^ However, strain-specific responses highlight the variability of biological systems beyond species-level differences. These data therefore support a condition-dependent effect of nanofiber architecture on biofilm formation, but they do not identify the underlying mechanism. Possible contributors may include differences in EPS production, surface chemistry, wettability, surface charge, and strain-specific cell-surface properties; however, these parameters were not measured in the present study and should therefore be regarded as hypotheses for future investigation.

Qualitative SEM analyses (Figures 5, 6) were carried out as supportive analyses to the quantitative results obtained by the CFU/cm^2^ enumeration method. Additional images, of both 48 h and 8 days cultures, are reported in Figures S1 and S2. This analysis further supported the quantitative results, showing that both LR strains easily adhered to PCL nanomaterials. Preferred attachment sites on the nanomaterial provide sufficient space for bridging fibers; when fibers are widely spaced, bridging is hindered in short-term cultivation but may diminish as a limiting factor during long-term cultivation.

**Figure 5:**
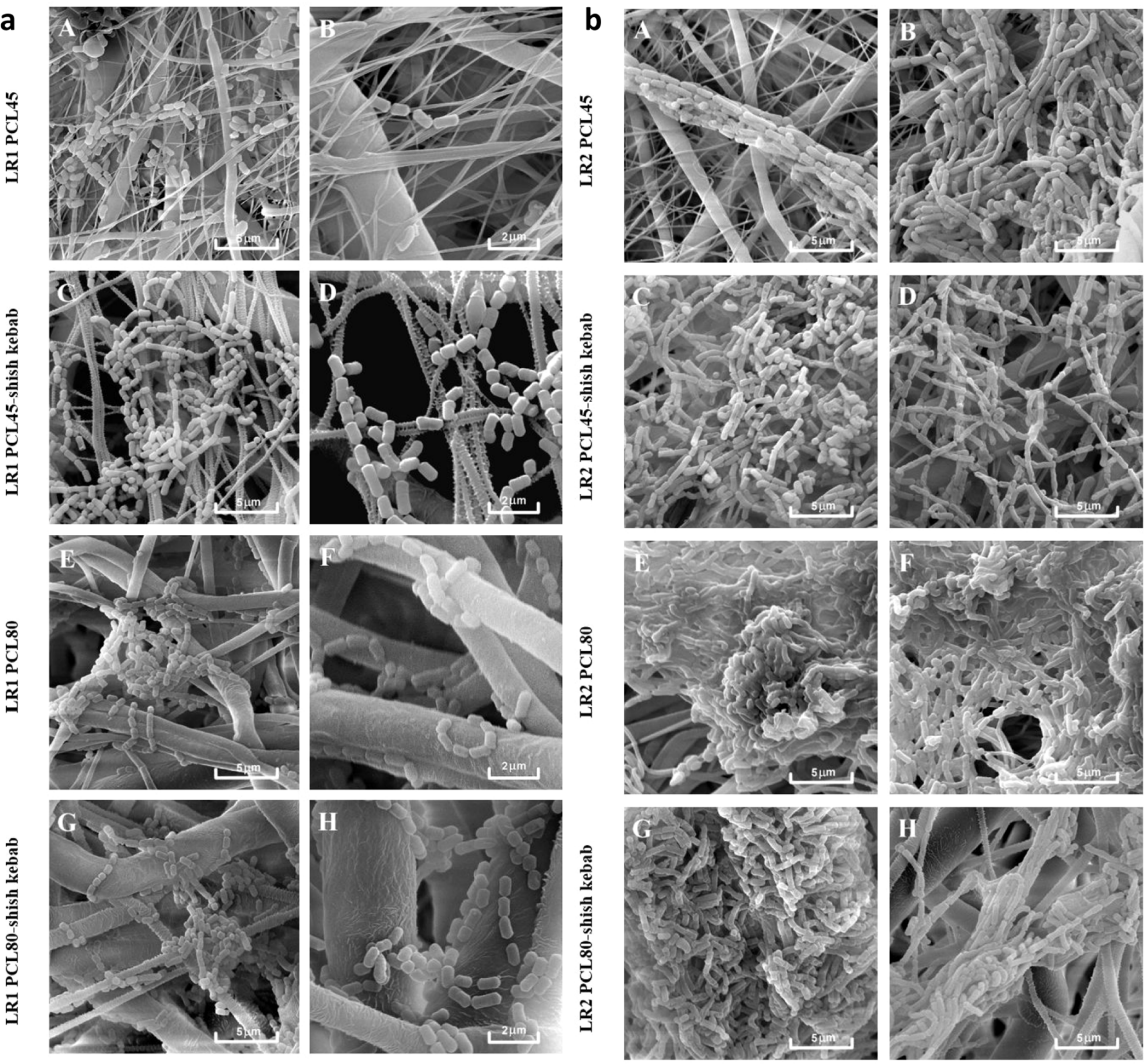
Qualitative analysis of LR1 (a) and LR2 (b) biofilm on the PCL nanomaterials (48 h) visualized by SEM—PCL45 (A, B), PCL45-sk (C, D), PCL80 (E, F), PCL80-sk (G, H).

**Figure 6:**
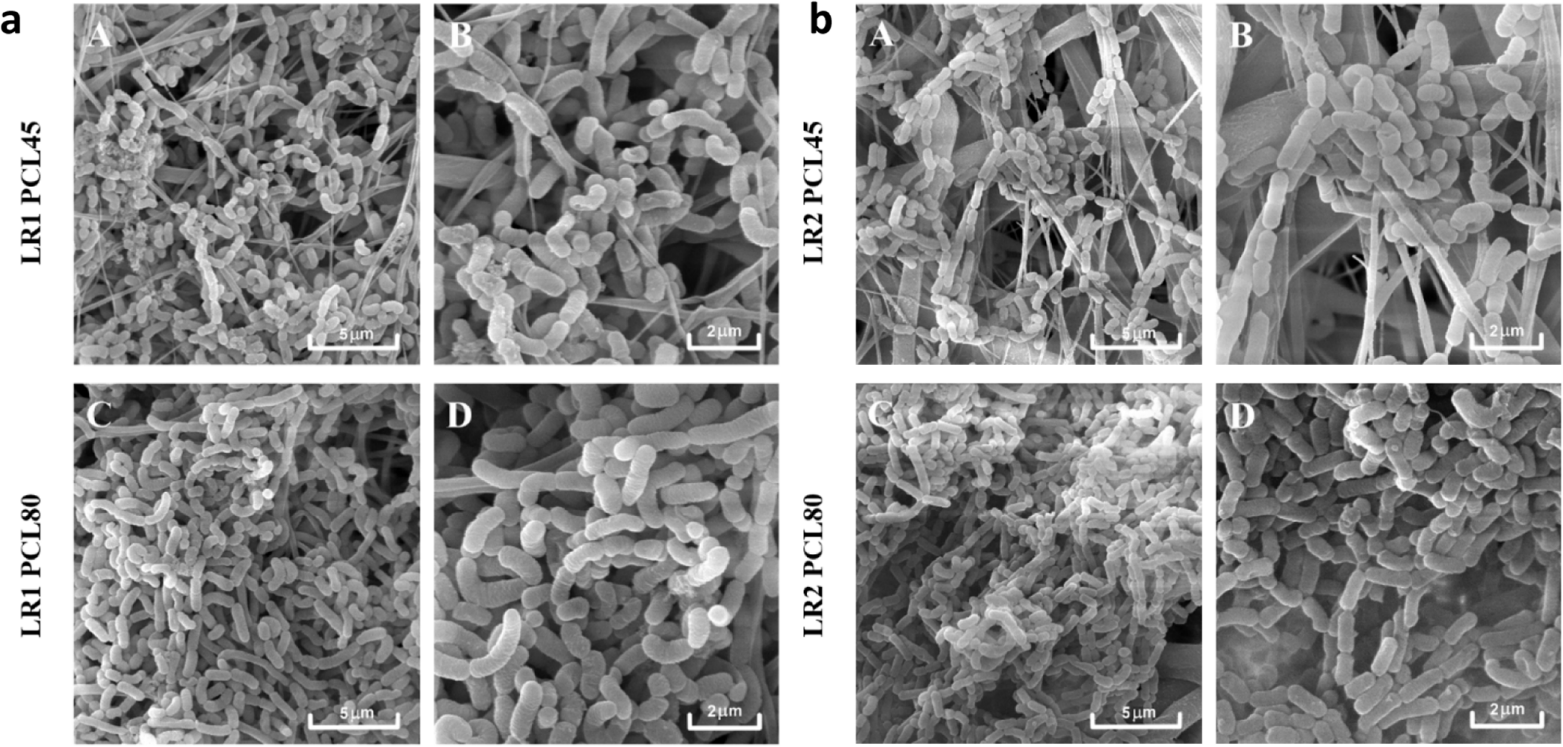
Qualitative analysis of LR1 (a) and LR2 (b) biofilm on the PCL nanomaterials (8 days) visualized by SEM—PCL45 (A, B), PCL80 (C, D).

These findings provide new insight into the field, as a limited number of studies investigating the impact of morphological parameters on microbial behavior were published. Regarding *sk* surface topography, no studies focusing on its impact on bacterial adhesion and biofilm formation are available, implying that the results can be discussed only based on the interim findings of our study. Although our results did not demonstrate a clear influence of the prepared surface structures on biofilm formation, it is reasonable to hypothesize that variations in structural parameters such as size, shape, and spatial distribution could yield markedly different outcomes. We propose that bacterial and microbial responses to *sk* structures are highly dependent on these specific attributes, similar to the way fundamental morphological parameters of nanofibers, such as fiber diameter, surface density, and porosity, influence their physicochemical and biological properties. However, it is important to note that the described observations reflect the specific strains, fabrication parameters, and 48 h/8 days cultivation window and should not be generalized beyond this experimental context without further investigation. Moreover, because only two laboratory *L. rhamnosus* strains were examined under controlled *in vitro* conditions, the present findings should be regarded as preliminary and not as universally applicable design principles for probiotic biofilm carriers. To advance our understanding in this area, future research should focus on the systematic fabrication of nanomaterials with well-defined surface architectures of varying geometries, followed by their evaluation using selected bacterial strains.

To further deepen our knowledge about the mutual interaction of PCL nanomaterials with LR, and following the quantitative results of biofilm formation, we determined the metabolic activity of the biofilm-forming cells. Numerous techniques have been employed to investigate metabolic activity and bacterial biofilm formation, with tetrazolium salt-based assays remaining widely used. Initially developed and characterized for mammalian cell cultures, these methods have since been extensively adapted for bacterial applications.^26^ Notably, biofilm metabolic activity is often spatially heterogeneous due to nutrient and oxygen gradients such that viable counts and bulk metabolic readouts may diverge. In this context, tetrazolium-based assays primarily report overall cellular redox activity rather than biomass *per se*, shifts in redox state or the biofilm microenvironment can modulate the readout independently of CFU.^39^ Given the established suitability of the MTT assay for analyzing biofilm metabolism,^40^ we employed this method to investigate potential changes in cellular metabolic activity following incubation with PCL nanomaterials.

To the best of our knowledge, no previous studies have assessed the metabolic activity of *L. rhamnosus* cultured on PCL-based substrates, providing initial insight into this issue. Our results (Figure 1c) revealed that, after 48 h of incubation, the metabolic activity of the LR1 strain remained relatively stable across all tested nanomaterials. In contrast, LR2 exhibited a marked decrease in metabolic activity after incubation with each of the four nanomaterial types. The observed decrease in MTT signal for LR2 indicates lower bulk metabolic/redox activity under the tested conditions compared with LR1. Because MTT primarily reflects overall reducing activity rather than direct viability or specific physiological states, these differences should not be interpreted as direct evidence of stress induction or dormancy. Instead, they indicate strain-dependent metabolic responses that may reflect differences in redox balance, substrate interaction, or biofilm physiology. When the incubation period was extended up to 8 days, both LR1 and LR2 exhibited a marked decrease in metabolic activity in the control samples (Figure 1d), suggesting that prolonged cultivation was associated with a metabolically reduced yet viable state—consistent with widespread observations that bacterial cells transition into lower-activity phenotypes during extended cultivation or environmental stress.^41^ Such metabolically downshifted subpopulations are widely described in mature biofilms and can contribute to stress tolerance even when culturability is maintained.^42^ In line with recent persister-focused work, low-growth subpopulations can also retain measurable metabolic and transcriptional activity despite limited division, supporting the possibility of reduced MTT signal without proportional loss of culturability.^43^ However, further mechanistic interpretation of the presented results would require complementary methods beyond MTT and CFU enumeration. Taken together, the divergence between CFU and MTT suggests that high numbers of culturable cells can coexist with reduced average bulk redox activity within the biofilm. This is consistent with the physiological heterogeneity of mature biofilms and/or lower per-cell metabolic activity during prolonged cultivation, but it does not by itself demonstrate dormancy or persistence. This leads to recommendations for further research, which should deeper investigate which culture conditions optimize metabolic activity, thereby enabling the full expression of the probiotic strains’ beneficial properties.

Based on the above findings, which are essential for understanding the potential of PCL nanomaterials as probiotic carriers, the following conclusions can be drawn, emphasizing differences in LR behavior depending on the cultivation approach (short/prolonged). **H1** “PCL nanomaterials enhance biofilm formation compared to PS” was accepted for 48 h cultivation, but after 8 days, differences between PS and PCL decreased, with strain-specific trends emerging. **H2** “Fiber diameter plays a significant role in promoting biofilm formation” and **H3** “Shish-kebab surface morphology of nanofibers influences the biofilm formation” were rejected for 48 h but accepted for 8 days of cultivation. This all implies highly strain- and condition-dependent behavior within these systems and underscores the need for further research to explore definitive trends and the underlying mechanistic explanations for this behavior. Also, we emphasize the need to assess not only viable cell counts but also metabolic activity, which decreases during prolonged cultivation.

### 3.3 Biofilm Resistance toward Different pHs

A key characteristic of probiotic strains is their ability to survive in acidic environments, a crucial trait for enduring the gastrointestinal tract (GIT) and exerting beneficial effects.^22^ Biofilm formation has been shown to enhance microbial resistance to extreme pH conditions, with both acidophilic and alkaliphilic species commonly found in such structures.^10^ *L. rhamnosus* GG, one of the most extensively studied probiotic strains, is recognized for its strong resistance to acidic and bile-rich conditions, as well as its robust growth, which supports its persistence in the GIT.^7,24^ However, no available data on the pH tolerance of LR1 have been reported in the literature. Furthermore, to our knowledge, no studies have investigated the potential acid resistance of *L. rhamnosus* biofilms in the presence of PCL nanomaterials. Therefore, this study aimed to explore whether LR1 and LR2 exhibited enhanced acid resistance when incubated with PCL nanofibers, compared to biofilms formed on a reference material, PS. The hypothesis **H4** “PCL-promoted biofilms possess high resistance to acidic pHs”, with an 80% survival criterion, was tested. Here, pH tolerance was assessed as a defined, short-time acid challenge (10 min of exposure) applied to preformed biofilms (48 h or 8 days) as an initial stress test, rather than as prolonged cultivation at the target pH. The selected pH values (2, 4, and 7) represent relevant conditions of the GIT environment, with pH 2 lying within the acidic range reported for the fasted human stomach and being the most challenging condition applied within this assay, whereas postprandial gastric pH is transiently higher, typically in the approximate range of pH 4–7.^44^ Accordingly, the reported CFU/cm^2^ values represent survival after exposure under the specific challenge duration used in this assay.

Our results (Figure 7) indicated generally high tolerance of formed biofilms to the tested pH conditions, with survival values mostly exceeding the predefined 80% criterion (resistance was evaluated based on CFU values and a comparison between PCL materials and the reference material, PS). This indicates that the tested *L. rhamnosus* strains are intrinsically tolerant to low pH under our assay conditions; PCL may provide an additional contribution to acid resistance under selected strain- and material-dependent conditions. However, the experiments yielded an inconsistent pattern regarding strain- and material-specificity. For both strains, biofilm viability (CFU/cm^2^) varied across all tested materials, regardless of fiber diameter or surface morphology, with only a few showing higher biofilm tolerance on PCL nanomaterials (Table S3). For LR1, at pH 2, the control yielded 7.7 ± 0.33 log_10_(CFU/cm^2^), while PCL nanomaterials supported a different biofilm viability, with a significant (*p* < 0.05) stimulatory effect observed only for PCL45 (8.4 ± 0.11 log_10_(CFU/cm^2^)). At pH 4, control viability was 8.2 ± 0.14 log_10_(CFU/cm^2^), and although differences were noted among PCL nanomaterials, no significant pH effect was detected. At pH 7, on the PS control, an average of 7.8 ± 0.14 log_10_(CFU/cm^2^) was revealed, while on the PCL nanomaterials a different biofilm viability was observed. In this case, only the PCL45 (8.3 ± 0.25 log_10_(CFU/cm^2^)) and PCL80 (8.8 ± 0.23 log_10_(CFU/cm^2^)) nanomaterials significantly enhanced the biofilm formation (Table S3A, S3B). For LR2, at pH 2 the control showed 6.2 ± 0.03 log_10_(CFU/cm^2^). On the nanomaterials different results were obtained, with a significant enhancement of biofilm observed in association with PCL80 nanomaterial (6.9 ± 0.18 log_10_(CFU/cm^2^)), while PCL80-sk showed a significant decrease (5.7 ± 0.22 log_10_(CFU/cm^2^)). At pH 4, control viability was 7.2 ± 0.24 log_10_(CFU/cm^2^). On the nanomaterials, a different viability was present, with PCL45 (6.9 ± 0.06 log_10_(CFU/cm^2^)) and its *sk* variant (6.7 ± 0.25 log_10_(CFU/cm^2^)) leading to significantly lower biofilm levels (Table S3C, S3D). At pH 7, the biofilm viability on the control was 7.3 ± 0.50 log_10_(CFU/cm^2^). Significant viability reductions were observed on PCL45-sk (6.4 ± 0.07 log_10_(CFU/cm^2^)) and PCL80-sk (6.4 ± 0.08 log_10_(CFU/cm^2^)). This generally indicates that in the case of specialized tests, it is necessary to consider a number of parameters and further research is crucial to develop pH-optimized probiotic formulations for food and pharmaceutical applications. Based on these results, the hypothesis **H4** “PCL-promoted biofilms possess high resistance to acidic pHs” was considered partially supported because both strains generally maintained high survival after the defined short-term pH challenge, whereas a significant PCL-associated enhancement relative to PS was observed only in selected strain/material combinations. However, its full acceptance or rejection would need further investigation.

**Figure 7:**
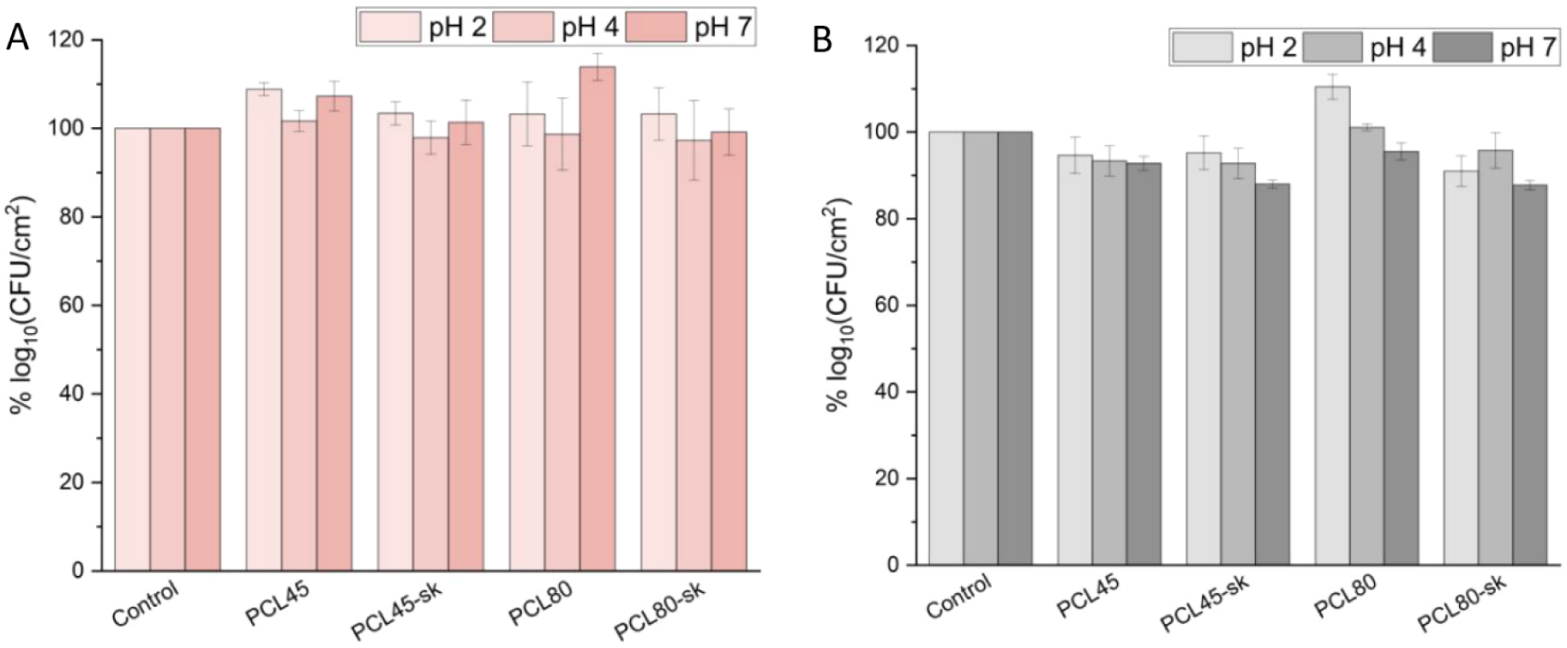
Resistance of LR1 (A) and LR2 (B) biofilm on smooth (PCL45, PCL80) and *sk* (PCL45-sk, PCL80-sk) nanomaterials to different pH levels (pH 2, pH 4, pH 7).

While the general pH tolerance of lactobacilli has been extensively studied, investigations into the pH resistance of their biofilms on nanomaterials are relatively recent and have emerged only in the past decade. Hu et al. reported that *L. paracasei* biofilms cultured on electrospun cellulose acetate nanofibrous membranes exhibited enhanced tolerance to harsh conditions, including strong acids, alkalis, and antibiotics.^8^ In a separate study, Hu et al. also reported that *L. plantarum* biofilms formed on cellulose acetate nanofibrous membranes exhibited greater *in vitro* gastrointestinal resistance (pH 2.5–6.8) compared to planktonic cells.^14^ Our results for LR1 generally align with previous studies, although not all nanomaterials and conditions led to an increased level of biofilm formation. In contrast, LR2 showed reduced biofilm production under the tested conditions, exhibiting the opposite trend. These discrepancies may stem from the limited literature on the pH resistance of bacterial biofilms on nanomaterials and the variability in the experimental conditions across studies. Therefore, further research is needed to better understand bacterial biofilm resistance to acidic environments, particularly in the context of PCL and other nanomaterials. In addition, to better assess physiological relevance, future studies should evaluate additional setups, including long-term low-pH exposure of mature biofilms.

### 3.4 Biofilm Resistance toward Microbial Contamination

For practical applications of PCL materials as *L. rhamnosus* biofilm carriers, it is important to keep the bacterial culture resistant against external contamination as much as possible. Several studies described the ability of *L. rhamnosus* strains (particularly *L. rhamnosus* GG) to inhibit/reduce *S. aureus* growth. Peng et al.^5^ revealed the antimicrobial activity of *L. rhamnosus* SCB0119 against *E. coli* and *S. aureus*. Mohammedsaeed et al.^45^ demonstrated the ability of *L. rhamnosus* GG to inhibit the toxic effect of *S. aureus* toward keratinocytes. Alhubail et al.^6^ demonstrated that neutralized supernatant of*L. rhamnosus* GG (as well as other probiotic bacteria) reduced the growth of *S. aureus* and other pathogenic microorganisms. Different works also reported the ability of *L. rhamnosus* species to reduce the viability of *Salmonella enterica* serovar Typhimurium, *S. aureus*, and *P. fluorescens* during *in vitro* studies. Furthermore, Jeong and Moon isolated a bacteriocin, rhamnocin 519, from *L. rhamnosus*, which demonstrated antimicrobial activity against *Listeria monocytogenes* and *S. aureus*.^7^ Despite these results, the ability of *L. rhamnosus* to inhibit pathogens still remains generic and thus cannot be guaranteed. Indeed, Prezzi et al.^46^ oppositely demonstrated that *L. rhamnosus* GG is not able to inhibit the growth of *S. aureus* in Minas Frescal cheese. Thus, antibacterial activity needs to be verified for each strain and culture conditions, which crucially influence the overall microbial activity. Following this knowledge, the hypothesis **H5** “PCL-associated biofilm exhibits enhanced resistance to *S. aureus* contamination compared to the biofilm formed on PS” was tested. Here, resistance refers to the relative survival of preformed LR biofilms after 24 h *S. aureus* challenge, expressed as survival percentage relative to the challenged PS control. Strain-specific responses were observed (Figure 8), with LR1 showing higher resistance to *S. aureus* contamination than LR2. This difference likely reflects strain-specific variation in competitive fitness and biofilm functionality during coculture, although these determinants were not directly measured in the present study. For LR1, the 48 h biofilm exhibited high survival (>80%) on PCL45, moderate survival (50–79%) in four combinations of biofilm cultivation and PCL type, and low survival (<50%) in three combinations, whereas all LR2 cultivation combinations showed low survival.

**Figure 8:**
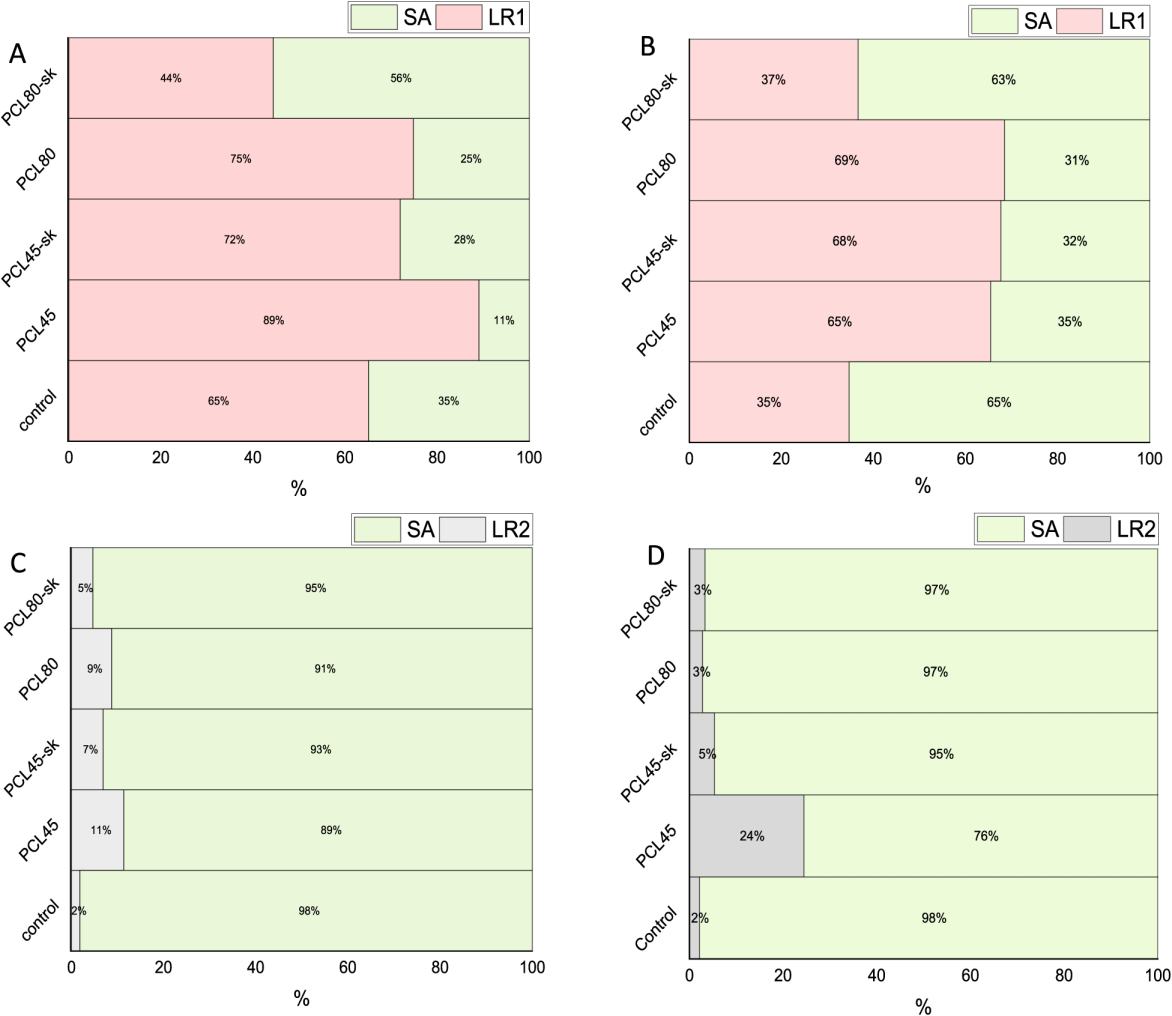
Resistance of LR1 (A, B) and LR2 (C, D) biofilm on smooth (PCL45, PCL80) and *sk* (PCL45-sk, PCL80-sk) nanomaterials to *S. aureus* contamination. A, C) 48 h matured biofilm; B, D) 8 days matured biofilm. The biofilm survival rate is reported as a percentage of biofilm related to the control well without nanomaterials.

Regarding LR1, our results (Figure 8A,B) supported the thesis of the above-mentioned studies.^5–7,45^ For LR1, the contamination assay indicated lower *S. aureus* recovery in association with PCL45 and PCL45-sk than with the thicker-fiber materials under the tested conditions (both after 48 h and 8 days of incubation). This observation may be attributed to the biofilm-forming capacity of *S. aureus* itself on PCL nanomaterials,^38^ or to nanofiber morphology, which may influence how two species compete for surface occupancy during the contamination challenge; that may be attributed to the comparable diameters between the fibers and the bacterium size (0.945 ± 0.671 μm for PCL80 nanofibers; 1 to 1.5 μm in length for *S. aureus* ^38^). However, this interpretation remains preliminary because species-specific attachment to individual materials was not directly quantified and no species-resolved imaging was performed. Therefore, we cannot conclude that *S. aureus* preferentially colonized the thicker fibers, and this explanation should be regarded as a working hypothesis for a future study.

Notably, our preliminary antimicrobial overlay assay yielded contrasting results (Table S4), indicating no detectable intrinsic anti-*S. aureus* activity for LR1 under those assay conditions. Because the antimicrobial assay and the contamination assay probe different phenomena—agar-based growth inhibition versus the outcome of *S. aureus* challenge of a preformed biofilm on a material surface—the results are not directly interchangeable. Nevertheless, the partial inconsistency between these assays indicates that any conclusion about the enhanced resistance of LR1 on selected PCL materials should be considered preliminary.

Regarding the LR2 strain, although the preliminary antimicrobial assay indicated that LR2 exhibited antimicrobial activity (Table S4), aligning with several published studies,^5–7,45^ the contamination assay conducted in the presence of PCL nanomaterials for 48 h (Figure 8C) produced results consistent with those reported by Prezzi et al.,^46^ suggesting that this strain does not exhibit a definitive antimicrobial effect against SA. These results were further supported by our investigation extending the incubation period of *L. rhamnosus* strains with the nanomaterials up to 8 days (Figure 8D), demonstrating that even with prolonged contact between the nanomaterials and LR2, no antimicrobial activity could be observed. This finding may be considered alongside the MTT assay results (Figure 4), which showed decreased bulk metabolic and redox activity of LR2 after 48 h and 8 days of incubation with all four types of nanomaterials. However, because MTT does not directly resolve viability, dormancy, or antimicrobial function, any mechanistic connection between the reduced MTT signal and antimicrobial compound production remains preliminary. Similar to LR1, this hypothesis-generating interpretation requires further targeted assays to be confirmed or rejected. It is worth noting that after a prolonged incubation period, the resistance of LR2 was enhanced by the presence of the PCL45 nanomaterial. This result suggests that the prolonged incubation period could allow the LR2 strain to exert its intrinsic antimicrobial activity also in the presence of a specific type of PCL nanomaterial.

SEM analysis of LR1 or LR2 biofilms (48 h, Figure 9; 8 days, Figure 10; Figures S3 and S4) cocultured with SA on all four types of nanomaterials supported the quantitative analysis, clearly showing the coexistence of LR strains and SA in biofilms, with varying representation on particular species.

**Figure 9:**
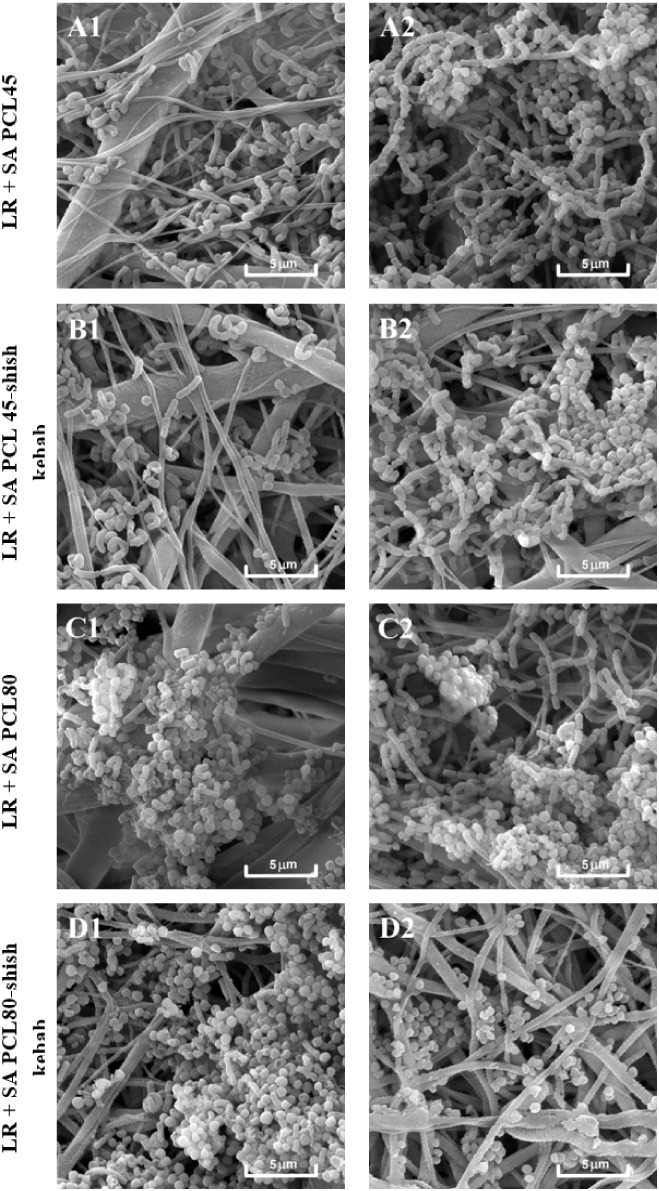
Qualitative analysis of LR biofilm on the PCL nanomaterials (48 h) in association with SA—visualized by SEM. LR1—A1, B1, C1, D1; LR2—A2, B2, C2, D2.

**Figure 10:**
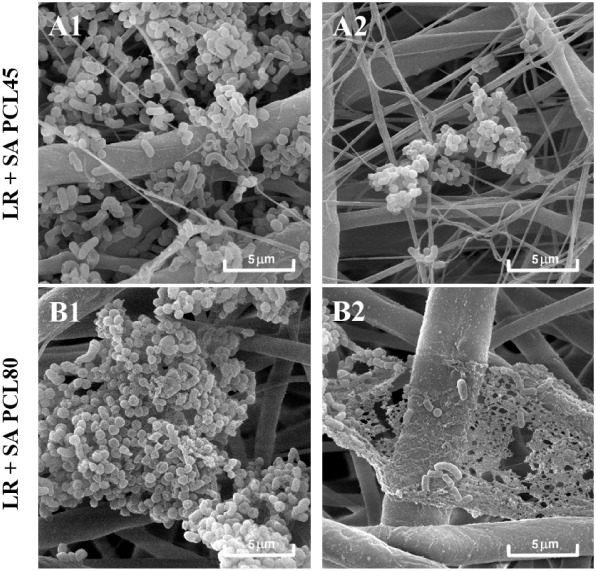
Qualitative analysis of LR biofilm on the PCL nanomaterials (8 days) in association with SA—visualized by SEM. LR1—A1, B1, C1, D1; LR2—A2, B2, C2, D2.

Based on the above-mentioned findings, the contamination assay suggests that LR1 biofilms on selected PCL materials may show improved resilience to *S. aureus* challenge relative to PS under the tested conditions; however, this observation is preliminary and mechanistic conclusions cannot be drawn from the present data. For LR2, this effect was not consistently observed. The hypothesis **H5** “PCL-associated biofilm exhibits enhanced resistance to *S. aureus* contamination compared to the biofilm formed on PS” was only partially supported and only under selected strain- and condition-dependent combinations, thus warranting further in-depth testing. For LR1, improved resistance was observed for some PCL materials relative to PS, whereas for LR2 this effect was not consistently observed. No uniform significant impact of fiber diameter or surface structure across tested materials was found. The present data suggest that structural optimization of nanofibers may influence both biofilm formation and resilience to external stressors, but this possibility requires direct validation in future studies. Such tunability could enable the adaptation of material properties to meet specific application requirements, particularly in systems where robust probiotic immobilization, process stability, and resistance to environmental stress are desirable.

From an application perspective, the present PCL system should be considered alongside established probiotic immobilization approaches used in food and nutraceutical research, including well-established microencapsulation systems,^47^ dried carrier-based preparations, and nanofibrous or cellulose-derived^14^ supports designed to improve probiotic survival during processing, storage, and gastrointestinal passage.^8^ In comparison with these systems, electrospun PCL nanofibers presented here offer a distinct advantage in their highly tunable architecture, enabling systematic control over fiber diameter and surface topography. This positions them as a complementary platform to previously reported cellulose acetate nanofiber systems used as reusable starter cultures for fermented milk^14^ and as biofilm-enriching probiotic scaffolds.^8^ At the same time, the present study does not yet establish direct technological applicability in foods. Although the continuous needleless electrospinning approach used here is, in principle, compatible with scalable material production, practical implementation will require dedicated evaluation of long-term storage stability, behavior in real food matrices, and process-compatible sterilization and packaging. This is particularly relevant because other electrospun probiotic systems have already shown improved thermal stability^48^ or long-term storage performance;^30^ however, research in this area remains limited. In addition, translation toward food or nutraceutical applications will require careful consideration of the regulatory status of both the probiotic strain and the carrier material in relation to the intended end use, including microorganism-specific food-safety frameworks. Therefore, the results presented here are best interpreted as defining a design-oriented platform for the future development of probiotic immobilization systems relevant to agricultural, food, and nutraceutical applications.

To summarize the obtained results, under the conditions tested, PCL nanomaterials proved to be promising candidates as tunable probiotic-biofilm carriers and as a platform concept for future food and nutraceutical immobilization systems. They promoted biofilm formation by *L. rhamnosus* strains as biofilm levels were consistently higher on PCL than on PS and exhibited high tolerance to strongly acidic conditions across the tested pH range.

Alongside these results, the study also generated testable hypotheses and directions for follow-up research. Morphology-related effects (including the presence of *sk* surface structures) and long-term cultivation responses (including metabolic activity trends) were strain- and condition-dependent and should be regarded as exploratory. The physicochemical drivers of these effects remain unresolved because key interfacial parameters were not measured in the present study. In addition, biofilms formed on PCL nanomaterials showed variable, strain- and material-dependent responses to *S. aureus* contamination. These findings indicate potential protective effects in selected conditions, but antimicrobial protection cannot yet be generalized and requires further targeted investigation. Therefore, given the still limited knowledge currently available in the field of lactobacilli–nanomaterial interactions, further systematic studies are required to disentangle the contributions of nanofiber architecture and cultivation regime and to determine whether the trends observed here remain valid across a broader diversity of probiotic strains and more application-relevant environmental conditions. Such studies should also evaluate storage stability and performance in application-relevant matrices, thereby informing the development of scalable and sustainable probiotic biofilm production and deployment strategies. Potential future applications include reusable starter systems for fermented products, storage-oriented probiotic carriers, or biofilm-based platforms for controlled probiotic biomass production.

## Supplementary Material



## Notes

Detailed statistical analysis (Table S1, Table S2, Table S3); additional SEM images of biofilm formation on tested nanomaterials (Figure S1, Figure S2, Figure S3, Figure S4); antimicrobial activity of LR1 and LR2 (Table S4) (PDF)

## Abbreviations used

CFU: colony forming unit
LR: *Lacticaseibacillus rhamnosus*
MRS: De Man–Rogosa–Sharpe medium
MTT: 3-(4,5-dimethylthiazol-2-yl)-2,5-diphenyl tetrazolium bromide
PCL: polycaprolactone
PBS: phosphate bufferd saline
PS: polystyrene
SA: *Staphylococcus aureus*
SEM: scanning electron microscopy
sk: shish-kebab
TSA: Tryptone soy agar
TSB: Tryptone soy broth
