# Supplementary material for: Impact of Fiber Diameter and Surface Topography of PCL Nanofibers on Lacticaseibacillus rhamnosus Biofilm Formation and Resistance
Source: J Agric Food Chem. 2026 May 12;74(20):15659–72. doi: 10.1021/acs.jafc.5c16509 (PMC13220305; doi:10.1021/acs.jafc.5c16509)

SUPPORTING INFORMATION

Impact of Fiber Diameter and Surface Topography of PCL Nanofibers on *Lacticaseibacillus rhamnosus* Biofilm Formation and Resistance

Elisa Andreani[1,2†], Vaclav Peroutka[1†], Eva Kuzelova Kostakova[3], Ema Chudobova[3], Pavel Kejzlar[4], Sarka Hauzerova[3], Kristyna Havlickova[3], Marta Stindlova[1], Jana Jiresova[5], David Lukas[3], Simona Lencova[1*]

[1] University of Chemistry and Technology Prague, Faculty of Food and Biochemical Technology, Department of Biochemistry and Microbiology, Technicka 5, 16628 Prague, Czech Republic
[2] University of Insubria, Department of Biotechnology and Life Sciences, Via Jean Henry Dunant 3, 21100 Varese, Italy
[3] Technical University of Liberec, Faculty of Science, Humanities and Education, Department of Chemistry, Studentska 1402/2, 46117 Liberec, Czech Republic
[4] Technical University of Liberec, Institute for Nanomaterials, Advanced Technology and Innovations, Department of Advanced Materials, Bendlova 1409/7, 460 01 Liberec, Czech Republic
[5] University of Chemistry and Technology Prague, Faculty of Chemical Engineering, Department of Physics and Measurements, Technicka 5, 16628 Prague, Czech Republic

[†]These authors contributed equally to this work.

*Corresponding author: Simona Lencova, lencovas@vscht.cz

Table S1: ANOVA analysis of log_{10}(CFU) of LR1 (A, B) and LR2 (C, D) biofilm development (48 h) on the nanomaterials and polystyrene (PS). Significance levels: $p < 0.001$ (very strong, ***); $p < 0.01$ (strong, **); $p < 0.05$ (statistically significant, *); $p > 0.05$ (not significant, n). α value was set at 0.05

A

**Comparison LR1 on PS vs nanomaterials**	**p value**
PS vs PCL45	0.0360 (*)
PS vs PCL80	0.0082 (*)
PS vs PCL45-shish kebab	0.0379 (*)
PS vs PCL80-shish kebab	0.0227 (*)

B

**Comparison between nanomaterials (LR1)**	**p value**
PCL45 vs PCL45-shish kebab	0.7739 (n)
PCL80 vs PCL80-shish kebab	0.3895 (n)
PCL45 vs PCL80	0.0716 (n)
PCL45-shish kebab vs PCL80-shish kebab	0.4660 (n)

C

**Comparison LR2 on PS vs nanomaterials**	**p value**
PS vs PCL45	0.0281 (*)
PS vs PCL80	0.0111 (*)
PS vs PCL45-shish kebab	0.0051 (**)
PS vs PCL80-shish kebab	0.0033 (**)

D

**Comparison between nanomaterials (LR2)**	**p value**
PCL45 vs PCL45-shish kebab	0.1698 (n)
PCL80 vs PCL80-shish kebab	0.1506 (n)
PCL45 vs PCL80	0.9338 (n)
PCL45-shish kebab vs PCL80-shish kebab	0.5496 (n)

Table S2: ANOVA analysis of $\log_{10}$(CFU) of LR1 (A, B) and LR2 (C, D) biofilm development (8 days) on the nanomaterials and polystyrene (PS). Significance levels: $p < 0.001$ (very strong, ***); $p < 0.01$ (strong, **); $p < 0.05$ (statistically significant, *); $p > 0.05$ (not significant, n). α value was set at 0.05

A

**Comparison LR1 on PS vs nanomaterials**	**p value**
PS vs PCL45	0.0532 (n)
PS vs PCL45-shish kebab	0.0417 (*)
PS vs PCL80	0.0212 (*)
PS vs PCL80-shish kebab	0.0616 (n)

B

**Comparison between nanomaterials (LR1)**	**p value**
PCL45 vs PCL45-shish kebab	0.0016 (*)
PCL80 vs PCL80-shish kebab	0.0418 (*)
PCL45 vs PCL80	0.0004 (***)
PCL45-shish kebab vs PCL80-shish kebab	0.0473 (*)

C

**Comparison LR2 on PS vs nanomaterials**	**p value**
PS vs PCL45	5E-08 (***)
PS vs PCL45-shish kebab	0.0006 (***)
PS vs PCL80	0.0489 (*)
PS vs PCL80-shish kebab	0.0107 (*)

D

**Comparison between nanomaterials (LR2)**	**p value**
PCL45 vs PCL45-shish kebab	7E-05 (***)
PCL80 vs PCL80-shish kebab	0.0442 (*)
PCL45 vs PCL80	0.0052 (*)
PCL45-shish kebab vs PCL80-shish kebab	0.0002 (***)

Figure S1: SEM images of LR biofilm formation (48 h) on different types of nanomaterials. LR1- A1, B1, C1, D1. LR2 – A2, B2, C2, D2. Magnifications: 2kx.

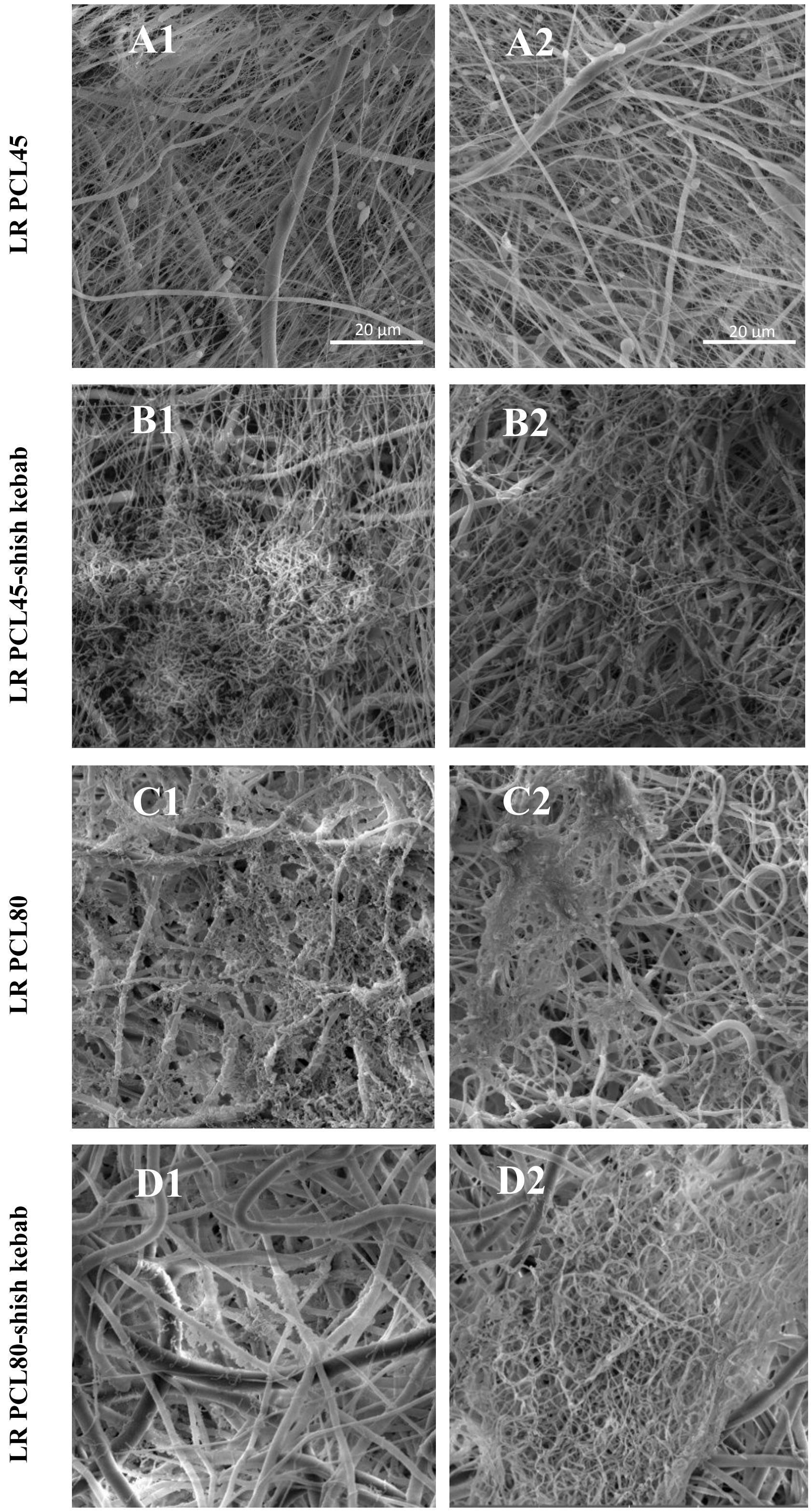

Figure S2: SEM images of LR prolonged (8 days) biofilm formation on different types of nanomaterials. LR1 - A1, B1. LR2 - A2, B2. Magnifications: 2kx.

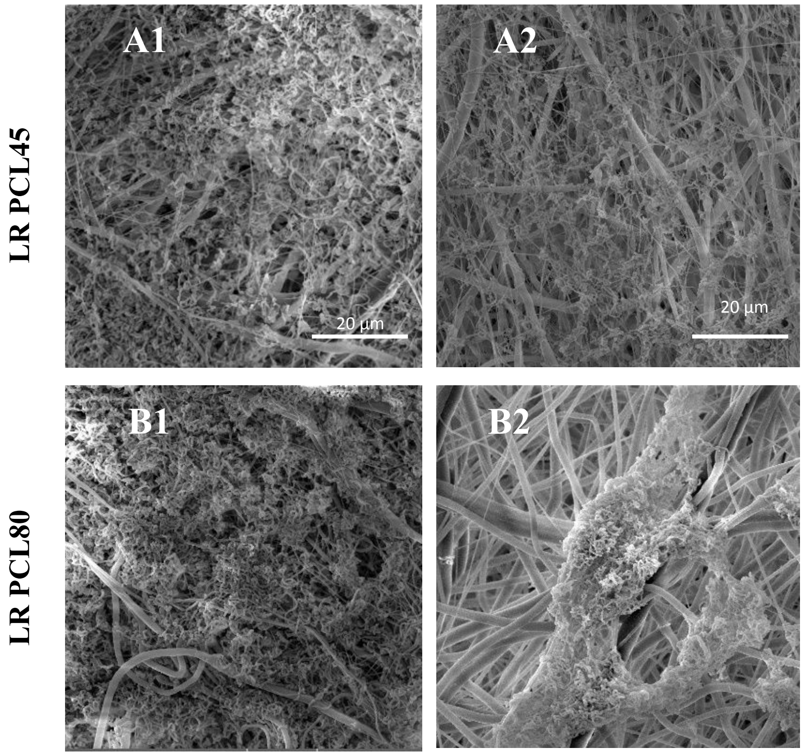

Table S3: ANOVA analysis of LR1 (A, B) and LR2 (C, D) biofilm development on different nanomaterials and PS at different pHs (2, 4, 7). Significance levels: $p < 0.001$ (very strong, ***); $p < 0.01$ (strong, **); $p < 0.05$ (statistically significant, *); $p > 0.05$ (not significant, n). α value was set at 0.05

A

pH level	PS vs PCL45	PS vs PCL45-shish	PS vs PCL80	PS vs PCL80-shish
pH 2	0.0071 (**)	0.1936 (n)	0.4482 (n)	0.3927 (n)
pH 4	0.3618 (n)	0.3963 (n)	0.7942 (n)	0.6289 (n)
pH 7	0.0081 (**)	0.0519 (n)	0.0001 (***)	0.7777 (n)

B

pH level	LR1 PCL45 vs PCL45-shish	LR1 PCL80 vs PCL80-shish	LR1 PCL45 vs PCL80	LR1 PCL45-shish vs PCL80-shish
pH 2	0.0131 (*)	0.9954 (n)	0.2326 (n)	0.9708 (n)
pH 4	0.1654 (n)	0.8500 (n)	0.5628 (n)	0.9066 (n)
pH 7	0.5432 (n)	0.0032 (**)	0.0452 (*)	0.1319 (n)

C

pH level	LR2 vs PCL45	LR2 vs PCL45-shish	LR2 vs PCL80	LR2 vs PCL80-shish
pH 2	0.0706 (n)	0.0683 (n)	0.0001 (***)	0.0020 (**)
pH 4	0.0169 (*)	0.0165 (*)	0.5766 (n)	0.1087 (n)
pH 7	0.1041 (n)	0.0162 (*)	0.1299 (n)	0.0150 (*)

D

pH level	LR2 PCL45 vs PCL45-shish	LR2 PCL80 vs PCL80-shish	LR2 PCL45 vs PCL80	LR2 PCL45-shish vs PCL80-shish
pH 2	0.8601 (n)	$2.4938 * 10^{-6}$ (***)	0.0002 (***)	0.1220 (n)
pH 4	0.8168 (n)	0.0426 (*)	0.0045 (**)	0.2529 (n)
pH 7	0.0045 (**)	$5.9943 * 10^{-5}$ (***)	0.0546 (n)	0.7937 (n)

Table S4: Antimicrobial activity of LR1 and LR2, determined via halo zones (mm).

	LR1		LR2	
**Sample**	**Halo zone (mm)**	**Colonies of *S. aureus* in the halo zone**	**Halo zone (mm)**	**Colonies of *S. aureus* in the halo zone**
1	0	Yes	18	No
2	0	Yes	16	No
3	0	Yes	30	No
4	0	Yes	25	No
5	0	Yes	25	Yes
6	0	Yes	25	Yes
Mean	0.0 ± 0.0		23.2 ± 4.7	

Figure S3: SEM images of LR strains growth (48 h) on different types of nanomaterials in association with SA. LR1 - PCL45 (A1), PCL80 (B1); LR2 - PCL45 (A2), PCL80 (B2). Magnifications: 2kx.

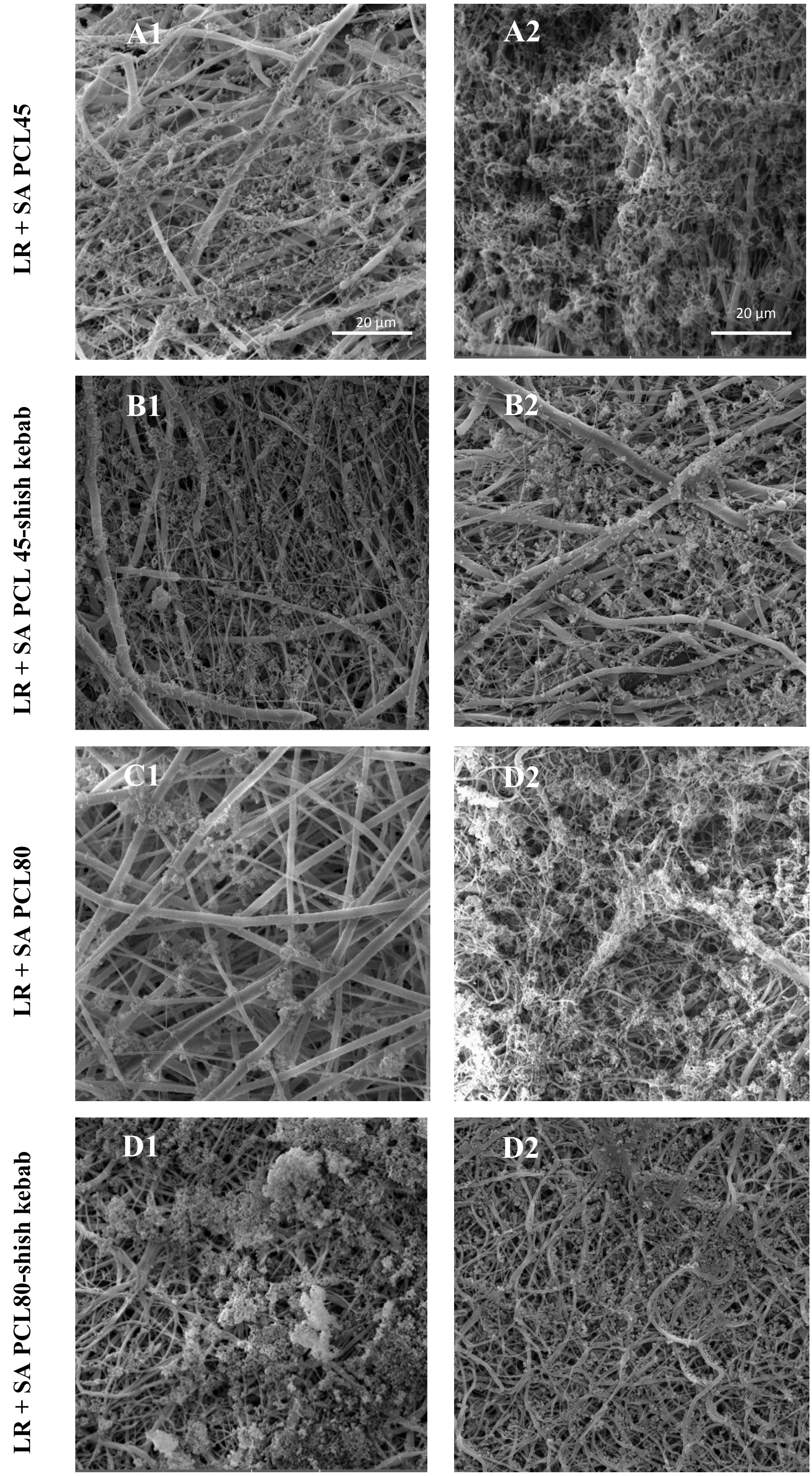

Figure S4: SEM images about LR strains growth on different types of nanomaterials in association with SA after a prolonged cultivation (8 days). LR1 - PCL45 (A1), PCL80 (B1); LR2 - PCL45 (A2), PCL80 (B2). Magnifications: 2kx.

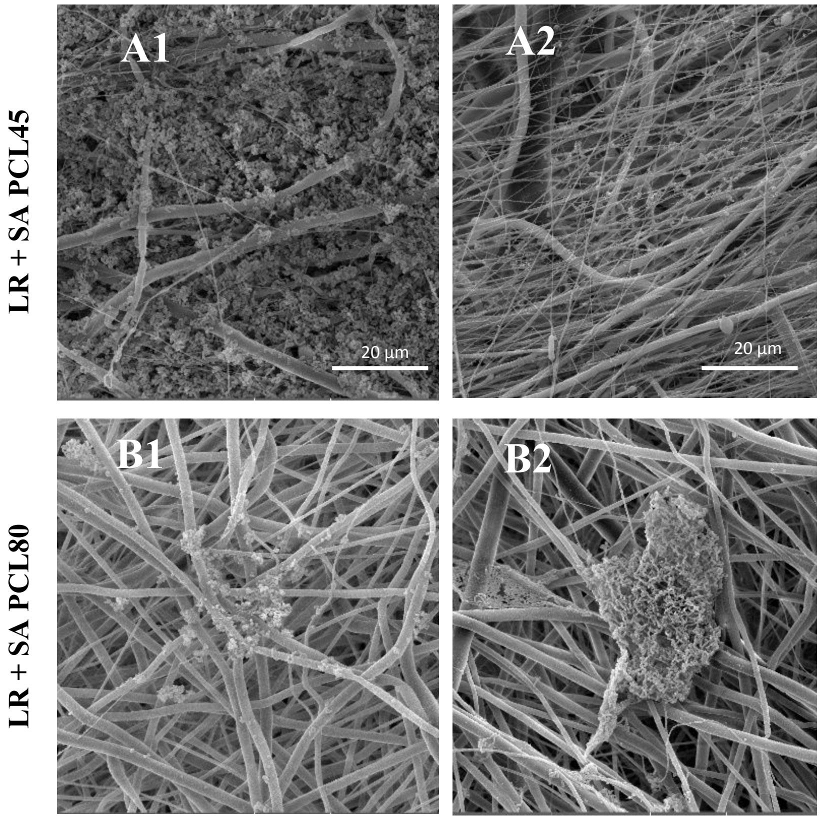

Supplement: Supplementary file 1 [file jf5c16509_si_001.pdf]
